# Analysis of Out-of-Hospital First Aid for Recovery of Spontaneous Circulation after Cardiac Arrest in Korea

**DOI:** 10.3390/diagnostics14020224

**Published:** 2024-01-20

**Authors:** Hyeon-Ji Lee, Mi-Young Choi, Young-Soon Choi

**Affiliations:** 1 Department of Emergency Medical Technology, College of Health Science, Kangwon National University, 346 Hwangjo-Gil, Samcheck-si 25949, Republic of Korea; 2 Department of Nursing, College of Health Science, Kangwon National University, 346 Hwangjo-Gil, Samcheck-si 25949, Republic of Korea

**Keywords:** out-of-hospital cardiac arrest, recovery of spontaneous circulation, first aid physical activity, paramedics

## Abstract

The characteristics of an individual patient experiencing out-of-hospital cardiac arrest who recovered spontaneous circulation with the assistance of witnesses and paramedics were examined. The analysis of bystander cardiopulmonary resuscitation (CPR) and the professional first aid efforts of paramedics in the pre-hospital environment is pivotal to enhancing the survival rate of out-of-hospital cardiac arrest patients. The data used in this study were extracted from the Korea Centers for Disease Control and Prevention (KCDC) nationally recognized statistics, Acute Heart Failure big data survey. Out-of-hospital cardiac arrest (OHCA) customer data were collected from the Gangwon Fire Headquarters public information database as social management data. The data were analyzed using SPSS 24. The study’s results emphasized the significance of offering basic CPR training to the public. This is evident from the fact that 90.5% of the first witnesses in the study performed CPR on OHCA patients, resulting in the recovery of spontaneous circulation (ROSC). The majority of patients with ROSC were male, with the highest age group being 41–50 years. Heart disease, hypertension, and diabetes were common medical conditions. The rate of witnessing cardiac arrest was high. Among the first witnesses, about 78.4% were of cardiac arrest incidents involving family members, co-workers, or acquaintances; 12.2% were on-duty medical healthcare personnel; and 9.5% were off-duty healthcare personnel. Cardiac arrest was treated in 83.8% of cases, with 90% of witnesses performing CPR. The percentage of witnesses that used an automated external defibrillator (AED) was 13.5%. In this study, the rates of ECG monitoring, CPR performance, and defibrillation performed by paramedics were high, but intravascular access and drug administration had a lower rate of performance. The time elapsed depended on the patient’s physical fitness. The study found that paramedics had the highest CPC restoration rate in patients with cardiac arrest, followed by EMTs and nurses. Significant differences were observed in cerebral performance scores after care by these paramedics and nurses. To increase the performance of AEDs, more AEDs should be installed in public spaces so that the public can access them conveniently in cases of emergency. In addition, it is necessary to improve the quality of professional first aid physical activity services performed by first-class paramedics.

## 1. Introduction

Out-of-hospital cardiac arrest (OHCA) is a significant health problem with an increasing prevalence and a reported incidence rate of 98 in 100,000 individuals in North America and 52 in 100,000 individuals in Asia [1]. The number of patients with OHCA is increasing in Korea, but the patients show a low survival rate and a discharge rate of <10% [2]. Cardiopulmonary resuscitation (CPR), an emergency treatment to resuscitate a patient with cardiac arrest, is the most basic and crucial emergency treatment and can increase the survival rate after a cardiac arrest. The patient’s chances of survival can be greatly increased if CPR is promptly administered by a witness during a cardiac arrest event. Rescue breaths and chest compressions are both part of CPR if the performer is trained. When the heart stops beating or is unable to pump blood efficiently, the goal is to manually pump blood and oxygen to essential organs. Survival rates drop dramatically if CPR is delayed [3,4,5]. Therefore, the performance of high-quality CPR and the appropriate application of a defibrillator are keys to the effective resuscitation of patients with OHCA [6,7,8]. According to previous studies, bystander cardiopulmonary resuscitation (CPR) was performed in approximately 51% of out-of-hospital cardiac arrest patients, and automated external defibrillation (AED) was performed in approximately 23%. When a cardiac arrest occurs, the survival rate increases when a witness performs CPR [9,10]. The rate of CPR performed by witnesses is gradually increasing. However, the use of automated external defibrillators has not increased as much as the use of CPR [10]. Recently, to increase the rate of bystander CPR, telephone-assisted CPR until the ambulance arrives is increasing. This is to motivate patients to perform CPR until the ambulance arrives and to instruct CPR by phone when they lack CPR experience or have not been trained [11]. Awareness of and education on CPR for patients with cardiac arrest are increasing. In addition, the availability and use of an automated external defibrillator are also important [11,12]. Automated external defibrillators are becoming widespread in public places, but they are in short supply, and it is often difficult to determine the installation location [9]. The use rate of AED for patients with cardiac arrest should be identified, and dissemination and education of AED should be supported. Because cardiac arrest is unpredictable, it is critical to the patient’s survival that the first witness performs CPR until an ambulance arrives. To facilitate the provision of emergency medical services (EMS), the Fire Department of Korea established emergency service providers in 1982 and an emergency treatment system that enabled paramedics to administer professional CPR at the site of a cardiac event [2,13]. Regarding patient survival, basic and professional CPR before hospital transfer is pivotal to increasing the rate of CPR administration [13,14]. As the initial response and treatment initiation are important in the prognosis of a patient with an acute cardiac arrest, national statistics of acute cardiac arrest have been used since 2011 [15].

In this study, data from patients who underwent out-of-hospital cardiac arrest and subsequently recovered spontaneous circulation (ROSC) were examined. The analysis specifically involved individuals transported to a hospital in Korea by ambulance. In particular, the results were analyzed by focusing on the identification of cardiac arrest by witnesses, whether basic CPR was performed, and the paramedics’ performance of advanced CPR. We confirmed whether paramedics performed high-quality CPR and specialized first aid at the scene. Factors affecting the patient’s survival rate were investigated through analysis of first aid performed by eyewitnesses and professional paramedics. Through this study, we witness cardiac arrest and emphasize the importance of basic CPR and advanced CPR. In addition, considering geographical characteristics, we propose a plan to increase the installation rate of automatic external defibrillators so that the general public can conveniently use them.

## 2. Materials and Methods

### 2.1. Research Subjects and Design

The research was conducted on 1476 subjects with cardiac arrest registered in the national statistical data of the Gangwon Fire Headquarters in the Republic of Korea from 1 January to 31 December 2019. Among them, the data of OHCA patients (*n* = 74) with ROSC before hospital arrival were analyzed. The dispatch log for patients with cardiac arrest, EMS operation log, and emergency treatment status log were used in this process. Because the data of the Gangwon Fire Headquarters are open to the public, no personally identifiable information was included in the study.

### 2.2. Research Area Emergency Medical System

The targeted area has a population density of 92.5 people and covers an area of 20,569 km^2^. The population aged 65 and older accounted for 302,886 of the total population of 1,560,571. Compared to other areas, the area chosen for this research has many rural areas and a larger elderly population. In addition, it is an area where emergency treatment in the field or in an ambulance is essential due to the long dispatch distance. The emergency medical system in the region is operated by 18 fire departments and uses 133 ambulances. Each ambulance is operated by a group of two or three people, including a paramedic or a nurse. When a request for medical support is registered at a fire station, the all-source situation center sends the nearest ambulance to the patient’s location. The center guides a witness to perform CPR over the phone and ensures that basic life support (BLS) is provided to the patient until the ambulance arrives. Once the ambulance arrives at the site of the emergency, a paramedic, if included in the team, provides high-quality advanced cardiovascular life support.

### 2.3. Statistical Analysis

The data required for this study were extracted from the Korea Centers for Disease Control and Prevention (KCDC) nationally recognized statistics, the Acute Heart Failure big data survey. OHCA customer data were collected from the Gangwon Fire Headquarters public information DB as social management data. The collected data included no personal information that could lead to the identification of the individuals. The daily log of emergency activities obtained from the fire station contains various aspects of emergency response. This crucial record contains the time and place where the patient was first discovered, information on the patient suffering from cardiac arrest (past history, current medical history, main symptoms, etc.), whether professional CPR was performed by paramedics, the status of first aid, if there were witnesses, bystander CPR, whether or not an AED was used, etc. By including these essential components, the log offers a comprehensive picture of the emergency response procedure, making it possible to conduct in-depth timeline analyses, integrate bystander interventions, and coordinate various aspects of emergency services. To start the emergency medical system, a witness discovers a patient suffering from cardiac arrest and activates the emergency medical system. The fire department performs CPR over the phone if the witness is capable of basic CPR. Therefore, in this study, it is verified whether CPR by paramedics and CPR by witnesses were performed well. The log likely follows established protocols for CPR, which may include the following general steps:

- Early identification of cardiac arrest based on clinical signs.
- Prompt activation of emergency services and dispatch of necessary resources.
- If trained witnesses are present, immediate initiation of high-quality CPR, including chest compressions and rescue breaths.
- When applicable, timely use of defibrillators for patients with shockable rhythms.
- Transition of care to EMS personnel upon their arrival, with the continuation of advanced life support measures.
- Descriptive statistics were analyzed using the SPSS ver. 24 (IBM Corp., Armonk, NY, USA) software package. Data were analyzed using the *t*-test and the analysis of variance test.

### 2.4. Ethical Considerations

The work was conducted according to the guidelines of the Declaration of Helsinki and approved by the Institutional Review Board of Kangwon University (Protocol Code: KWNUIRB-2021-11-003).

## 3. Results

### 3.1. General Characteristics of the Patients with ROSC

The majority of the patients were male. The percentage of patients with ROSC was highest in the age group of 41–50 years, followed by those of 51–60 years and 61–70 years. The distribution of the places of cardiac arrests was 54.1% public areas, 36.5% nonpublic areas, and 9.5% inside the ambulance. The medical history of the patients included heart disease in 27%, hypertension in 18.9%, and diabetes or other afflictions in 8.1%, The rate of witnessing cardiac arrest was high (83.8%) [Table 1].

### 3.2. Basic First Aid for Witness

The characteristics of the witnesses who discovered the cardiac arrest patients were investigated. In 78.4% of the events, the witnesses were family members, co-workers, or acquaintances, in 12.2%, they were on-call medical personnel, and in 9.5%, they were off-duty medical personnel. Cardiac arrest was observed in 83.8% of the cases, whereas it was not observed in 9.5% of the cases. A total of 6.8% of the incidents remained unknown. In 90% of the cases, the witness performed CPR, and in 5.4% of the cases, the witness did not perform CPR. The remaining 4.1% of the events are unknown with regard to the witness performing CPR. Witnesses did not use AEDs in 86.4% of the incidents. There are two possible reasons for this. The first possibility is that defibrillators were either unavailable or had restricted access, which would explain the lack of AED use (missing). On the other hand, the type of cardiac rhythm—that is, if it was considered non-shockable—might have an impact on the decision to forego using AEDs. Moreover, non-use indicates non-availability or a non-shockable rhythm in the absence of clear information on the causes. Regarding the provision of medical guidance before the arrival of the ambulance, 66.2% of the cases received medical guidance from the Comprehensive Situation Center, and 4.1% received medical guidance from paramedics [Table 2].

### 3.3. Difference in the Rate of CPR Performance of Witnesses According to the Location of Cardiac Arrest

As a result of examining whether or not witnesses performed CPR according to the location of the cardiac arrest, bystander CPR was highest at 92.6% when the cardiac arrest was discovered in a non-public place and was at 87.5% in a public place. There was no statistically significant difference (*p* > 0.05) [Table 3].

### 3.4. Differences in the Rate of Automatic External Defibrillation Performed by Witnesses According to the Location of Cardiac Arrest

As a result of looking at the differences in the rates of automatic external defibrillation performed by witnesses according to the location of cardiac arrest, 96.3% of cases did not perform the AED in non-public places, and 90.0% of cases did not perform it in public places. The performance rate in the ambulance was high at 71.4%. There was a statistically significant difference (*p* = 0.000) [Table 4].

### 3.5. Differences in Performing Basic CPR for Cardiac Arrest Patients According to Eyewitnesses

Related workers at work scored 3.00 points, the general public scored 2.86 points, and related workers not at work scored 2.71 points, with no statistically significant differences. The use of an automatic external defibrillator by witnesses was as follows: on-duty workers had 2.67 points, off-duty workers had 2.43 points, and the general public had 2.02 points. There was a statistically significant difference (*p* = 0.000). There was a statistically significant difference in medical guidance before ambulance arrival in the order of 2.52 points for the general public, 2.43 points for related workers not on duty, and 1.33 points for related workers on duty (*p* = 0.001) [Table 5].

### 3.6. General Characteristics of the EMS and the Analysis of Emergency Treatment

Among the general characteristics of the EMS, the inclusion of leading professional care providers among the EMS staff in an ambulance was as follows: a paramedic in 58.1% of the events, an EMT in 28.4% of the events, and a nurse in 13.5% of the events. The number of staff that boarded the ambulance was two in 74.3% of the events and three in 25.7% of the events.

The emergency treatment performed by the paramedics was considered a multi-response problem. Defibrillation using an automated external defibrillator (AED) was performed in 100% of the events. CPR was administered in 98.6% of the events. Here, CPR involves a combination of chest compressions and rescue breaths and is a fundamental component of resuscitation during a cardiac arrest. Chest compressions alone (without ventilation) was performed in 97.3% of the events, AED shock was performed in 83.8%, advanced airway management was performed in 33.8%, intravascular access was performed in 13.5%, and epinephrine administration was performed in 6.8%.

The first finding from the AED monitoring was ventricular fibrillation (VF) in 82.4% of the events; non-VF cardiac arrest in 5.4%; unknown in 4.1%; bradycardia in 2.7%; asystole, tachycardia, or normal sinus rhythm in 1.4%; and malfunction of the defibrillator in 1.4%. Defibrillation was performed zero times in 16.2%, one time in 37.8%, two times in 17.6%, three times in 14.9%, four times in 5.4%, five times in 4.1%, six times in 2.7%, and seven times in 1.4% of the events [Table 6]. In Table 6, Ventricular Fibrillation (VF), Ventricular Tachycardia (VT), Asystole, Pulseless Electrical Activity (PEA), and Bradycardia are relevant cardiac rhythms associated with cardiac arrest situations. When cases marked as bradycardia are searched for in the notation log written by the paramedics, there are two cases in which CPR was performed until the defibrillator was attached, but because the pulse returned when the rhythm was checked, they were analyzed as bradycardia.

### 3.7. Time Elapsed before Professional Emergency Treatment after Cardiac Arrest

**Figure 1:**
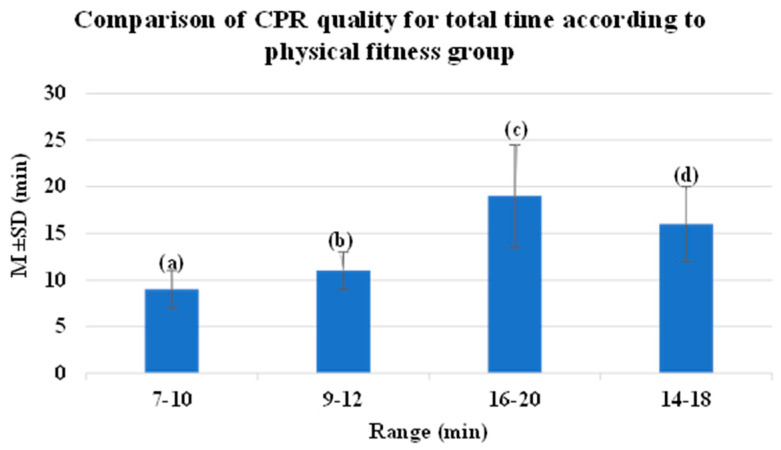
Block diagram of CPR quality for total time according to physical fitness group.

The average time for witness-directed CPR for a cardiac arrest patient was approximately 8.5 min. The average time for paramedics to perform advanced CPR was 10.5 min. It took approximately 18 min for the patient to arrive at the hospital. The average duration of CPR was 16 min [Figure 1], [Table 7].

### 3.8. Difference in the Cerebral Performance Restoration Rates of Patients with Cardiac Arrest Based on the Levels of the Leading Paramedics

CPC calculations were conducted at the emergency medical center. The fire department was later notified. The Cerebral Performance Category (CPC) is generally used to obtain neurological outcomes in survivors of cardiac arrest. It is measured on a scale from 1 to 5, where a lower score indicates better neurological function. From Table 8, it can be seen that paramedics with an average CPC score of 1.10 ± 0.53 had the highest restoration rate, followed by EMTs (1.39) and nurses (1.19). The differences in CPC scores among paramedics, EMTs, and nurses were found to be statistically significant (*p* > 0.01). The comparison between paramedics and nurses showed a *p*-value of 0.060, which is close to the significance threshold of 0.05. EMTs had an average CPC score of 1.39 ± 0.73. Nurses had an average CPC score of 1.90 ± 0.52. Further, statistically significant differences were observed in the cerebral performance scores after the provision of care by level 1 paramedics and nurses [Table 8].

## 4. Discussion

The emergency treatment received by patients with OHCA with ROSC was investigated by considering the factors associated with ROSC. Among the general characteristics of the patients with ROSC, the majority were males aged between 41 and 50 years. Middle-aged patients have higher physical capacities than older patients and thus have a better recovery rate after cardiac arrest. A cited paper comparing cardiac arrest outcomes between the elderly and young adults was reported [16].

Heart attacks in 54.1% of the cases occurred in public. Because this research included only patients with ROSC after cardiac arrest, the rate of cardiac events occurring in public places was high. Similar results have been reported in previous studies conducted worldwide [17,18,19,20]. When a cardiac arrest occurs in a public place, there are likely to be many witnesses. If the first witness performs CPR, which reduces the risk of heart failure, the patient is more likely to have a good prognosis. The percentage of eyewitnesses who first performed CPR on ROSC patients was as high as 90.5%. Other previous studies have also reported that eyewitness CPR increases survival and may even affect resuscitation rates [10,21,22]. Therefore, basic CPR by eyewitnesses is very important for the resuscitation of cardiac arrest patients. Standardized CPR training is essential for witnesses to perform CPR.

The percentage of witnesses using a defibrillator was 13.5%. This highlights the critical importance of early defibrillation in the event of cardiorespiratory arrest. It is still crucial to address issues that prevent people from using AEDs. According to eyewitnesses, there are several reasons for the low use rate of automatic external defibrillators. Some of them are that witnesses could not use AEDs because there were no automatic external defibrillators nearby and/or because of lack of training. The time-sensitive nature of interventions is emphasized by identifying the rhythm of the arrest, especially rhythms such as ventricular fibrillation or ventricular tachycardia. The initial rhythm of the cardiac arrest patient was ventricular fibrillation. This demonstrates that many patients present with a rhythm that can utilize defibrillation. Some previous studies have shown that early defibrillation can increase patient survival [23,24,25]. Previous studies have shown that survival rates increase when AEDs are installed and used in public places [5,26,27]. The area covered by this study includes many areas vulnerable to emergency medical services and many rural areas. Accordingly, increasing the deployment rate of AEDs in public places will bring positive results in shockable rhythm before the arrival of paramedics, which will further contribute to the patient’s survival. To increase the performance of defibrillation, more defibrillators should be installed in public spaces, so that the public can access them conveniently in cases of emergency. Moreover, because the percentage of the public who receive medical guidance over the phone before the arrival of an ambulance is high, an effort to develop a standardized training program for those using a phone is recommended. National and global studies have been conducted on how the performance of CPR by a witness receiving guidance over a phone call improves the survival outcomes of patients with OHCA [24]. Fundamentally, witness CPR and AED use go hand in hand, with early application of both greatly increasing the likelihood of survival. Investigating any observed behavioral shifts between the first witness and conventional witnesses is important because it could be an indication of advancements in accessibility and education.

It is important to ensure an environment where the provision of appropriate treatment, namely defibrillation, is possible. In previous studies, the early ECG of the resuscitated patients demonstrated high percentages of VF and tachycardia [25]. If a defibrillator is used during the early phase, when the ECG shows a rhythm in which defibrillation is possible, the resuscitation rate may increase. Regarding the leading professional care providers in the EMS ambulance, paramedics were included in 58.1% of the events. The number of EMS staff who boarded the ambulance was two in 74.3% and three in 25.7% of the events. In Korea, it is mandatory to include a minimum of two people in an ambulance; however, recently, the EMS staff number has been increased to ensure the inclusion of at least three people in the ambulance. The rate of ROSC is higher when a paramedic is present in the ambulance [28,29,30]. However, the emergency treatment that can be performed on a patient can be more diverse during three-person CPR than with two-person CPR. Recently, BLS and ACLS training for emergency medical workers has been changed to team training [31,32]. This talks about the importance of team CPR, and, ultimately, to perform team CPR in the field, standardized professional training of two or more people is required. A paramedic is needed. The specifics of EMS (emergency medical services) training and protocols are described as anatomy, physiology, and emergency medical procedures, which are often covered in the foundational training that EMTs receive. Basic life support (BLS) training includes patient assessment, CPR (cardiopulmonary resuscitation), and first aid. Paramedics receive more extensive training that includes knowledge of both advanced life support (ALS) and basic life support (BLS). Medication administration, advanced airway management, ECG (electrocardiogram) interpretation, and more complex medical procedures are all covered in advanced training. Emergency medical services (EMS) personnel receive training in standardized resuscitation procedures that adhere to global standards, including those set forth by the International Liaison Committee on Resuscitation (ILCOR). The treatment of respiratory distress, cardiac arrest, and other potentially fatal situations is covered by protocols. They also receive training in evaluating and treating trauma patients, which includes stopping bleeding, stabilizing fractures, and tending to seriously injured people right away. The use of vital medical equipment, such as defibrillators, airway management tools, and monitoring equipment, is also covered in training. To guarantee seamless patient care, EMS crews are trained in efficient communication and coordination with other medical specialists, hospitals, and pertinent authorities. EMS workers need to continue their education to stay current on emerging medical procedures and technology. Opportunities for professional development and regular training sessions are usually offered.

If an ambulance has a minimum of three staff members onboard, professional emergency services can be administered early to a patient both at the emergency site and during the transfer to a hospital. The professional emergency treatment provided by the paramedics is an important factor in increasing the chance of resuscitation in patients with cardiac arrest. Moreover, the rates of AED monitoring, CPR performance, chest compression, and defibrillation performance were high, whereas the performance of intravascular access and drug administration were low. This was because two people were sent to the emergency sites, and the number of paramedics who could provide various professional emergency services was low. In Korea, the number of people with the skills to provide professional emergency treatment is low, and there is a lack of legal information on providing emergency treatment over a phone call and administering drugs. Other previous studies also support this claim of insufficient skilled workforce; as a result, there has been a recent trend to increase the number of ambulances with at least three staff onboard.

The average time between the occurrence of a cardiac arrest and the provision of professional CPR by paramedics was 10.5 min. The time parameter is a crucial factor in the resuscitation rate of patients with cardiac arrest globally, and countries with developed EMS, such as the United States and Japan, take approximately 6 min to provide BLS and 7–10 min to provide defibrillation, with the professional CPR provided on-site influencing the ROSC [18,33].

When the CPC scores of the patients with cardiac arrest were compared based on the leading professional care providers, the score was highest in the case of paramedics. This finding shows that the range of possible professional emergency treatment depends on the level of the paramedic, thus making it an important factor for patients with cardiac arrest. The extensive scope of work of the paramedics regarding the provision of professional emergency services in an ambulance improves the resuscitation rate of patients globally [18,34].

This study only covered a specific region of Korea. An instruction manual for the treatment of patients with cardiac arrest should be prepared by procuring a greater paramedic workforce and deploying additional level 1 paramedics. The minimum number of paramedics riding in an ambulance to provide specialized first aid in the pre-hospital environment should be three, and the deployment of first-level emergency medical technicians with professional qualifications should be increased. This is based on a study analyzing France’s emergency medical system that found that survival rates can be increased by increasing the proportion of qualified emergency technicians and increasing the performance of prehospital CPR, emergency drug administration, and intravenous injections. The study shows similar results [35]. Moreover, the work scope of and the quality of professional emergency procedures performed by the level 1 paramedic should be enhanced. The occurrence of cardiac arrest is one of the emergencies that require basic treatment by a witness and professional emergency care by a team of paramedics. Such actions can improve the quality of emergency treatment before hospitalization and increase the resuscitation rate of patients with cardiac arrest.

## 5. Conclusions

The characteristics of patients with cardiac arrest due to spontaneous circulation and the general characteristics of first-aid physical activity provided by witnesses and para-medics were analyzed. Patients with cardiac arrest who recovered spontaneous circulation had a very high rate of CPR performed by witnesses. A high percentage of medical guidance was provided by fire department telephone counselors to encourage witnesses to perform CPR before the arrival of paramedics. However, the implementation rate was low as there were many cases where there were no automatic external defibrillators nearby. CPR by witnesses has become widespread, but the installation rate of automatic external defibrillators must be increased through publicity and various government policies. In addition, to increase the performance rate of CPR by phone consultation, if video phone-assisted CPR training is conducted and a standardized program is developed and disseminated, high-quality CPR can be provided. As a result of analyzing the first aid provided by paramedics, the deployment rate of paramedics with professional qualifications is determined to be only 58.1%, so the deployment rate must be increased to ensure that specialized first aid can be performed on patients. Additionally, the number of paramedics on board was high, with two people on board. In rural areas and areas with many emergency medical services vulnerabilities, such as in this study, three-person riding should be required to reduce the burden of first aid on paramedics and reduce paramedics’ fatigue. In addition, paramedics with professional qualifications should be deployed and professional education should be promoted to increase the performance rate of professional resuscitation.

## Figures and Tables

**Table 1:** General characteristics of the patients with ROSC.

Variables	Category	Frequency (*n*)	Percentage (%)
Gender	Female	9	12.2
	Male	65	87.8
Age (years)	0–20	1	1.4
	31–40	3	4.1
	41–50	23	31.1
	51–60	19	25.7
	61–70	13	17.6
	71–80	12	16.2
	≥80	2	2.7
	Unknown	1	1.4
Place of cardiac arrest	Public place	40	54.1
	Nonpublic place	27	36.5
	Inside the ambulance	7	9.5
Medical history	Hypertension	14	18.9
	Diabetes	6	8.1
	Heart disease	20	27
	Others	6	8.1
Witnessed cardiac arrest	Witnessed	62	83.8
	Not witnessed	7	9.5
	Unknown	5	6.8

**Table 2:** Basic first aid for witnesses.

Category		Frequency (*n*)	Percentage (%)
Relationship with the patient	Public (family members, colleagues, acquaintances, etc.)	58	78.4
	On-duty healthcare professionals	9	12.2
	Off-duty healthcare professionals	7	9.5
Cardiopulmonary resuscitation by a witness	Unknown	3	4.1
	Not performed	4	5.4
	Performed	67	90.5
Use of AED by a witness	Not performed	64	86.5
	Performed	10	13.5
Provision of medical guidance before the arrival of an ambulance	Did not receive any	22	29.7
	Paramedic	3	4.1
	Comprehensive Situation Center	49	66.2

**Table 3:** Difference in the rate of CPR performance of witnesses according to the location of cardiac arrest.

General Characteristics	Category	CPR by Witnesses			Total 100.0 (74)
		Unknown	Not Performed	Performed	
Place of cardiac arrest	Public place	7.5	5.0	87.5	100.0 (40)
	Nonpublic place	0	7.4	92.6	100.0 (27)
	Inside the ambulance	0	0	10.4	100.0 (7)
χ^2^ = 3.263, df = 4, sig = 0.515.					

**Table 4:** Differences in the rate of automatic external defibrillation performed by witnesses according to the location of cardiac arrest.

General Characteristics	Category	AED by Witnesses		Total 100.0 (74)
		Not Performed	Performed	
Place of cardiac arrest	Public place	90.0	10.0	100.0 (40)
	Nonpublic place	96.3	3.7	100.0 (27)
	Inside the ambulance	28.6	71.4	100.0 (7)
χ^2^ = 22.735, df = 2, sig = 0.000.				

**Table 5:** Differences in performing basic CPR for cardiac arrest patients according to eyewitnesses.

Types of Emergency Treatment	Relationship with the Patient	Emergency Treatment (M ± SD)	F	*p*	Post Hoc
CPR by witnesses	General public	2.860 ± 0.437	0.802	0.453	
	On-duty healthcare professionals	3.000 ± 0.000			
	Off-duty healthcare professionals	2.710 ± 0.756			
Implementation of an AED by a witness	General public ^A)^	2.020 ± 0.131	29.866	0.000	A:B, A:C
	On-duty healthcare professionals ^B)^	2.670 ± 0.500			
	Off-duty healthcare professionals ^C)^	2.430 ± 0.535			
Provision of medical guidance before the arrival of an ambulance	General public ^A)^	2.520 ± 0.843	7.745	0.001	A:B, B:C
	On-duty healthcare professionals ^B)^	1.330 ± 0.707			
	Off-duty healthcare professionals ^C)^	2.430 ± 0.976			

**Table 6:** General characteristics of the EMS and the analysis of emergency treatment.

Category		Frequency (*n*)	Percentage (%)
Leading professional care providers	Paramedic	43	58.1
	EMT *	21	28.4
	Nurse	10	13.5
Number of people in the ambulance	2	55	74.3
	3	19	25.7
Emergency treatment (multiple answers)	Chest compression	72	97.3
	ECG * monitoring	74	100
	AED * shock	62	83.8
	Intravascular access	10	13.5
	Administration of epinephrine	5	6.8
	Others	1	1.4
	Maintenance of airway	25	33.8
Initial electrocardiogram rhythm	VF *	61	82.4
	PVT *	1	1.4
	Asystole	1	1.4
	Pulseless electrical activity	4	5.4
	Bradycardia	2	2.7
	Normal	1	1.4
	Unknown	3	4.1
	Error	1	1.4
Total number of defibrillations	0	12	16.2
	1	28	37.8
	2	13	17.6
	3	11	14.9
	4	4	5.4
	5	3	4.1
	6	2	2.7
	7	1	1.4

* EMT = emergency medical technician, ECG = electrocardiogram, AED = automated external defibrillator, VF = Ventricular. Fibrillation, PVT = Pulseless Ventricular Tachycardia.

**Table 7:** Time elapsed before professional emergency treatment after cardiac arrest.

Category	M ± SD (min)	Range (min)
(a) Time elapsed before the administration of professional cardiopulmonary resuscitation after the registration of the first report	00:09 ± 00:04	7–10
(b) Time elapsed before the administration of professional defibrillation after the registration of the first report	00:11 ± 00:04	9–12
(c) Time taken to reach the hospital after the registration of the first report	00:19 ± 00:11	16–20
(d) Time elapsed before resuscitation after the registration of the first report	00:16 ± 00:08	14–18

**Table 8:** Different CPC rates of patients with cardiac arrest based on the levels of the leading professional care providers.

General Characteristics	Category	Cerebral Performance Score	F	*p*	Post Hoc
Leader Paramedics	Paramedics ^A)^	1.10 ± 0.53	2.93	0.060	A:C
	EMT ^B)^	1.39 ± 0.73			
	Nurses ^C)^	1.90 ± 0.52			

## Data Availability

There are restrictions on the availability of these data. Data were obtained from the Korea Gangwon Fire Department National Statistics.
